# Human Stem Cells in Disease Modelling and Treatment: Bridging the Gap Between Bench and Bedside

**DOI:** 10.3390/biomedicines13092313

**Published:** 2025-09-22

**Authors:** Alvaro Plaza Reyes, Sofia M. Calado

**Affiliations:** 1 Department of Integrative Pathophysiology and Therapy, Andalusian Molecular Biology and Regenerative Medicine Centre (CABIMER), 41092 Seville, Spain; 2 Faculty of Sciences and Technology, University of Azores-Polo de Ponta Delgada, Rua da Mãe de Deus, 9500-321 Ponta Delgada, Portugal

**Keywords:** human stem cells, disease modeling, regenerative medicine, organoids, cell therapy, clinical translation, mesenchymal stem cells, induced pluripotent stem cells, ethical guidelines, biomanufacturing

## Abstract

Human stem cell research is entering a stage where disease modeling, translational applications, and clinical therapies are increasingly connected. This editorial provides an overview of the contributions included in this Special Issue, titled “Human Stem Cells in Disease Modelling and Treatment”, placing them within the wider landscape of stem cell science. We summarize advances in ovarian stem cells for infertility, mesenchymal stem cells for neurodegeneration, pluripotent stem cell-derived cardiovascular and kidney organoids, adipose-derived stem cells, and emerging immunomodulatory and neural progenitor approaches. These studies illustrate the breadth of stem cell research and its potential to inform clinical practice. At the same time, challenges remain in reproducibility, safety, scalability, and ethical oversight. Looking forward, collaborative work and harmonized global standards will be important to bring laboratory findings into therapies that are safe, effective, and accessible. This editorial closes the first edition of the Special Issue with a reflection on current progress and directions for the future.

## 1. Introduction

The field of human stem cell research has undergone a remarkable transformation over the last two decades, transitioning from pioneering discoveries in developmental biology to clinical applications that now shape the future of regenerative medicine. The advent of human embryonic stem cells (hESCs), followed by induced pluripotent stem cells (iPSCs), provided unprecedented access to patient-specific and pluripotent cell sources capable of differentiating into virtually any cell type. This breakthrough, coupled with advances in organoid culture systems, genome editing technologies, and bioengineering, has positioned stem cell-based approaches as a cornerstone for disease modeling, drug development, and therapeutic innovation [1,2].

At the same time, the limitations of traditional preclinical animal models have become increasingly evident. Rodents, despite their utility, often fail to capture key aspects of human physiology and disease. This disconnect contributes to high dropout rates in drug development pipelines, as promising interventions fail in human clinical trials due to species-specific differences in genetics, immune responses, or organ physiology [3]. The ability to generate human tissue models and transplantable grafts directly from patient-derived stem cells therefore represents a transformative opportunity to improve translational fidelity, increase therapeutic success rates, and reduce the reliance on animal models.

This Special Issue of *Biomedicines*, titled “Human Stem Cells in Disease Modelling and Treatment”, brings together contributions that highlight the breadth of applications for human stem cells across diverse fields. The published articles collectively showcase advances in reproductive biology, neurology, cardiology, nephrology, immunology, and regenerative medicine. In this editorial, we provide an integrative overview of these contributions, contextualize them within the broader landscape of human stem cell research, highlight persistent challenges, and identify promising future directions. By doing so, we aim to underscore both the scientific and clinical momentum of this rapidly evolving field.

## 2. From Animal Models to Human-Relevant Systems

Biomedical research has long depended on animal models to investigate disease mechanisms and to test therapeutic candidates. However, the translational gap between rodent models and human clinical trials is stark, with a significant proportion of drug candidates failing due to unpredicted toxicities or inefficacies. Human stem cell-derived models, particularly iPSC-derived organoids and tissues, offer an alternative strategy that more accurately reflects human-specific biology. These systems enable the recapitulation of patient-specific genotypes and phenotypes, as well as tissue-specific cellular heterogeneity [4].

The ability to generate organoids, self-organizing, three-dimensional tissue structures derived from stem cells, has been especially transformative. Organoids recapitulate aspects of tissue architecture and function, and their emergence has opened new frontiers in disease modeling for neurological disorders, congenital heart disease, polycystic kidney disease, and cancer [5]. Furthermore, the advent of assembloids, complex systems that combine multiple organoid types or tissue lineages, has allowed the modeling of inter-organ interactions, such as brain–muscle or brain–vascular connectivity.

Complementing organoid technology, CRISPR-Cas9 gene editing allows for precise manipulation of disease-associated mutations, enabling researchers to create isogenic control lines for disease modeling [6]. This approach strengthens causal inference and has already been used to model conditions such as cystic fibrosis, amyotrophic lateral sclerosis, and Duchenne muscular dystrophy. Together, organoid systems and gene editing technologies exemplify the maturation of human stem cell platforms into robust and predictive disease models.

## 3. Highlights from the Special Issue

The seven articles included in this Special Issue reflect the diversity of applications for stem cells in biomedical research and clinical translation.

1. Ovarian Stem Cells in Infertility
  Grettka et al. (2024) examined ovarian stem cells (OSCs) as a potential therapeutic avenue for women with premature ovarian failure (POF) or cancer treatment-related infertility (CTRI) [7]. Conventional fertility preservation strategies, such as cryopreservation, are not always feasible. OSCs, with the capacity to differentiate into oocyte-like cells (OLCs), present a new horizon in reproductive medicine. The authors discuss challenges in OSC characterization, differentiation efficiency, and ethical considerations while emphasizing the clinical potential of OSCs for patients who currently lack therapeutic alternatives.
2. Mesenchymal Stem Cells in Spinocerebellar Ataxia
  Lee et al. (2024) reviewed the therapeutic potential of mesenchymal stem cells (MSCs) for spinocerebellar ataxia (SCA), a group of progressive neurodegenerative diseases [8]. MSCs exert immunomodulatory and neurotrophic effects, and preclinical studies suggest that they can enhance Purkinje cell survival and motor coordination. Although preliminary clinical data indicate safety and feasibility, the authors underscore the need for randomized controlled trials to determine long-term efficacy and optimize delivery protocols.
3. Cardiovascular Organoids for Disease Modeling
  Stougiannou et al. (2024) discussed the utility of pluripotent stem cell-derived cardiovascular organoids to study cardiac development, congenital heart disease, and drug-induced cardiotoxicity [9]. These models provide valuable insight into cardiomyocyte maturation and tissue-level electrophysiology. Despite these advances, the field continues to grapple with limitations in vascularization and structural maturation [10]. Bioengineering strategies, including microfluidic platforms and electrical stimulation, hold promise for improving physiological fidelity [11].
4. Kidney Organoids for Polycystic Kidney Disease
  Scarlat et al. (2025) explored the application of kidney organoids to model autosomal dominant polycystic kidney disease (ADPKD) [12]. Organoids carrying PKD1 or PKD2 mutations display cyst formation reminiscent of patient pathology, providing a robust system for mechanistic studies and therapeutic screening [13]. This work highlights the importance of organoids as preclinical platforms for diseases with limited therapeutic options.
5. Adipose-Derived Stem Cells and Tissue Collection Conditions
  Pal et al. (2024) investigated how ischemic versus vascularized adipose tissue collection influences the biological properties of adipose-derived stem cells (ADSCs) [14]. They reported that ischemic harvesting reduces adipogenic differentiation and triglyceride storage, thereby impacting ADSC functional potential. This finding underscores the importance of tissue procurement and handling for the quality of stem cell-derived products.
6. Human Umbilical Di-Chimeric Cells for Immunomodulation
  Siemionow et al. (2024) introduced human umbilical di-chimeric cells (HUDCs) generated via ex vivo fusion of donor umbilical cord blood cells [15]. HUDCs demonstrated engraftment and biodistribution in immunocompromised mice without tumorigenicity, suggesting potential as immunomodulatory therapies for transplantation tolerance and autoimmune disease management.
7. Ovarian Cortical-Derived Neural Progenitors for Parkinson’s Disease
  González-Gil et al. (2025) presented evidence for the neural differentiation capacity of ovarian cortical-derived progenitors under the influence of follicle-stimulating hormone (FSH) [16]. Their ability to generate dopaminergic neurons with electrophysiological activity points to unconventional sources for autologous therapies in Parkinson’s disease.

## 4. Lessons from Recent Clinical Case Studies

Stem cell-based therapies are gradually moving from concept to clinical reality. Landmark trials with human pluripotent stem cell-derived retinal pigment epithelium (RPE) for macular degeneration demonstrated both anatomical integration and signs of functional stabilization [17,18]. Similarly, recent first-in-human studies with dopaminergic neuron progenitors in Parkinson’s disease have shown safety and cell survival, with preliminary functional improvements [19].

These trials underscore the importance of rigorous product characterization, careful patient selection, and long-term monitoring. They also highlight the need for standardization of potency assays, validated manufacturing protocols, and harmonized regulatory frameworks to ensure that stem cell therapies are both safe and effective.

## 5. Challenges and Knowledge Gaps

Despite these promising results, human stem cell research continues to face persistent challenges that must be addressed for the field to realize its full clinical potential. One major hurdle is the lack of standardization in differentiation protocols. Even for the same lineage, methods can vary significantly between laboratories, leading to inconsistent results and limiting reproducibility [20]. Recent benchmarking efforts using high-dimensional molecular profiling have highlighted the extent of this variability and underscored the need for harmonized quality standards across laboratories [21]. This variability complicates comparisons across studies and underscores the need for harmonized protocols and rigorous benchmarking.

In addition, these systems frequently remain developmentally immature, displaying fetal-like gene expression profiles, electrophysiological activity, or metabolic states. Such immaturity affects not only their structural and functional fidelity but also their ability to respond to stimuli in a manner comparable to adult tissues [22]. As a result, disease models may capture early-onset or developmental phenotypes more accurately than late-onset or degenerative conditions, and drug testing platforms may fail to predict adult-specific pharmacological responses or toxicities. Addressing this limitation requires the development of maturation strategies, such as prolonged culture, biomechanical stimulation, vascularization, and co-culture with supporting cell types, that more closely approximate the adult in vivo environment.

Safety also remains a central concern. The risks of genomic instability, acquired during prolonged culture or as a consequence of reprogramming, pose barriers to clinical application [23]. Moreover, although iPSCs were initially thought to be immunologically inert, subsequent studies revealed that even autologous iPSC derivatives can trigger immune responses under certain conditions [24]. Addressing these safety issues requires stringent quality control and regulatory readiness, requiring potency assays, long-term biodistribution monitoring of transplanted cells, and careful assessment of tumorigenic potential.

Scalability and manufacturing are additional obstacles. Translating stem cell technologies into widely available clinical therapies requires robust, cost-effective, and cGMP-compliant production systems. Current workflows are often labor-intensive and difficult to scale, raising concerns about feasibility for widespread clinical use [25]. Recent works have demonstrated that identifying extracellular markers for lineage-specific enrichment can streamline differentiation workflows and open the door to automation, thereby increasing reproducibility and scalability [26]. Furthermore, advances in automation, closed culture systems, and high-throughput bioprocessing will be critical to overcome these barriers.

Finally, ethical and regulatory considerations remain at the forefront of stem cell research. The field continues to navigate questions related to the use of embryonic stem cells, gene editing, and the commercialization of stem cell therapies. Gametogenesis from stem cells raises sensitive questions about reproductive autonomy, genetic identity and parenthood, and the possibility of germline modification, all of which require open societal dialogue and regulatory guidance [27]. Global harmonization of ethical guidelines, such as those issued by the ISSCR, is essential for maintaining public trust and ensuring responsible innovation [28].

## 6. Future Directions

Looking ahead, several exciting avenues are poised to shape the next phase of human stem cell research. The first involves the development of more physiologically complex and mature organoid models. Incorporating vascular networks, immune cells, and extracellular matrix components will bring organoids closer to in vivo conditions, enhancing their utility for disease modeling and therapeutic screening.

Advances in gene editing are also expected to play a transformative role. Base editing and prime editing technologies offer precise correction of single nucleotide variants and larger genomic alterations with reduced off-target effects [29,30]. These tools will enable the generation of more accurate disease models and, in the longer term, support the development of curative therapies for genetic disorders.

Bioengineering innovations will further accelerate progress. The integration of organ-on-chip platforms, 3D bioprinting, and microfluidic systems allows for the modeling of tissue–tissue interactions, drug delivery, and mechanical stimulation [31]. These hybrid systems bridge the gap between organoids and whole-organ physiology, offering unprecedented control and resolution in experimental design.

The role of artificial intelligence (AI) and multi-omics cannot be overstated. AI-driven approaches will facilitate the integration of complex single-cell transcriptomic, epigenomic, and proteomic datasets, enabling the prediction of differentiation outcomes and the identification of novel biomarkers [32]. Such computational tools will guide the rational design of differentiation protocols and therapeutic strategies.

Finally, the success of future clinical translation will depend on collaborative networks. Large-scale consortia, public–private partnerships, and global data-sharing initiatives are necessary to establish standardized best practices and to accelerate the path from discovery to therapy [33]. The convergence of interdisciplinary expertise, including stem cell biology, genetics, bioengineering, computational biology, and clinical medicine, will define the next chapter in this field [34].

## 7. Conclusions

The Special Issue, “Human Stem Cells in Disease Modelling and Treatment”, illustrates the dynamic trajectory of stem cell research, spanning basic discovery, disease modeling, translational platforms, and early clinical applications. The progress on stem cell research depends on platforms that are human-relevant, functionally mature, standardized, and ethically grounded. While these significant barriers remain, the collective evidence points to a future where human stem cells become central tools in both understanding and treating human disease. With continued innovation, rigorous validation, and responsible regulation, stem cell-based approaches have the potential to reshape medicine and bring lasting benefits to patients worldwide.

## Abbreviations

The following abbreviations are used in this manuscript:

hESCs	Human embryonic stem cells
iPSCs	Induced pluripotent stem cells
POF	Premature ovarian failure
CTRI	Cancer treatment-related infertility
OLCs	Oocyte-like cells
MSCs	Mesenchymal stem cells
SCA	Spinocerebellar ataxia
ADPKD	Autosomal dominant polycystic kidney disease
ADSCs	Adipose-derived stem cells
HUDCs	Human umbilical di-chimeric cells
FSH	Follicle-stimulating hormone
RPE	Retinal pigment epithelium
cGMP	Current good manufacturing practice
ISSCR	International Society for Stem Cell Research
AI	Artificial intelligence

## References

[1] Takahashi K., Tanabe K., Ohnuki M., Narita M., Ichisaka T., Tomoda K., Yamanaka S. (2007). Induction of Pluripotent Stem Cells from Adult Human Fibroblasts by Defined Factors. Cell.

[2] Clevers H. (2016). Modeling Development and Disease with Organoids. Cell.

[3] Shi Y., Inoue H., Wu J.C., Yamanaka S. (2017). Induced Pluripotent Stem Cell Technology: A Decade of Progress. Nat. Rev. Drug Discov..

[4] Lancaster M.A., Knoblich J.A. (2014). Organogenesis in a Dish: Modeling Development and Disease Using Organoid Technologies. Science.

[5] Revah O., Gore F., Kelley K.W., Andersen J., Sakai N., Chen X., Li M.Y., Birey F., Yang X., Saw N.L. (2022). Maturation and Circuit Integration of Transplanted Human Cortical Organoids. Nature.

[6] Hsu P.D., Lander E.S., Zhang F. (2014). Development and Applications of CRISPR-Cas9 for Genome Engineering. Cell.

[7] Grettka K., Idzik K., Lewandowska K., Świętek K., Palini S., Silvestris F. (2024). Ovarian Stem Cells for Women’s Infertility: State of the Art. Biomedicines.

[8] Lee G.B., Park S.M., Jung U.J., Kim S.R. (2024). The Potential of Mesenchymal Stem Cells in Treating Spinocerebellar Ataxia: Advances and Future Directions. Biomedicines.

[9] Stougiannou T.M., Christodoulou K.C., Karangelis D. (2024). In Vitro Models of Cardiovascular Disease: Embryoid Bodies, Organoids and Everything in Between. Biomedicines.

[10] Ronaldson-Bouchard K., Ma S.P., Yeager K., Chen T., Song L., Sirabella D., Morikawa K., Teles D., Yazawa M., Vunjak-Novakovic G. (2018). Advanced Maturation of Human Cardiac Tissue Grown from Pluripotent Stem Cells. Nature.

[11] Bhatia S.N., Ingber D.E. (2014). Microfluidic Organs-on-Chips. Nat. Biotechnol..

[12] Scarlat A., Tomasoni S., Trionfini P. (2025). Autosomal Dominant Polycystic Kidney Disease: From Pathogenesis to Organoid Disease Models. Biomedicines.

[13] Czerniecki S.M., Cruz N.M., Harder J.L., Menon R., Annis J., Otto E.A., Gulieva R.E., Islas L.V., Kim Y.K., Tran L.M. (2018). High-Throughput Screening Enhances Kidney Organoid Differentiation from Human Pluripotent Stem Cells and Enables Automated Multidimensional Phenotyping. Cell Stem Cell.

[14] Pal P., Medina A., Chowdhury S., Cates C.A., Bollavarapu R., Person J.M., McIntyre B., Speed J.S., Janorkar A.V. (2024). Influence of the Tissue Collection Procedure on the Adipogenic Differentiation of Human Stem Cells: Ischemic versus Well-Vascularized Adipose Tissue. Biomedicines.

[15] Siemionow M., Chambily L., Brodowska S. (2024). Efficacy of Engraftment and Safety of Human Umbilical Di-Chimeric Cell (HUDC) Therapy after Systemic Intraosseous Administration in an Experimental Model. Biomedicines.

[16] González-Gil A., Rojo C., Ramírez E., Martín R., Suárez-Pinilla A.S., Ovalle S., Ramos-Ruiz R., Picazo R.A. (2025). Proneurogenic Actions of FSH During Directed Differentiation of Neural Stem and Progenitor Cells from Ovarian Cortical Cells Towards the Dopaminergic Pathway. Biomedicines.

[17] Schwartz S.D., Hubschman J.-P., Heilwell G., Franco-Cardenas V., Pan C.K., Ostrick R.M., Mickunas E., Gay R., Klimanskaya I., Lanza R. (2012). Embryonic Stem Cell Trials for Macular Degeneration: A Preliminary Report. Lancet.

[18] Kashani A.H., Lebkowski J.S., Rahhal F.M., Avery R.L., Salehi-Had H., Dang W., Lin C.-M., Mitra D., Zhu D., Thomas B.B. (2018). A Bioengineered Retinal Pigment Epithelial Monolayer for Advanced, Dry Age-Related Macular Degeneration. Sci. Transl. Med..

[19] Tabar V., Sarva H., Lozano A.M., Fasano A., Kalia S.K., Yu K.K.H., Brennan C., Ma Y., Peng S., Eidelberg D. (2025). Phase I Trial of HES Cell-Derived Dopaminergic Neurons for Parkinson’s Disease. Nature.

[20] Volpato V., Webber C. (2020). Addressing Variability in IPSC-Derived Models of Human Disease: Guidelines to Promote Reproducibility. Dis. Model Mech..

[21] Zhao C., Plaza Reyes A., Schell J.P., Weltner J., Ortega N.M., Zheng Y., Björklund Å.K., Baqué-Vidal L., Sokka J., Torokovic R. (2025). A Comprehensive Human Embryo Reference Tool Using Single-Cell RNA-Sequencing Data. Nat. Methods.

[22] Papamichail L., Koch L.S., Veerman D., Broersen K., van der Meer A.D. (2025). Organoids-on-a-Chip: Microfluidic Technology Enables Culture of Organoids with Enhanced Tissue Function and Potential for Disease Modeling. Front Bioeng. Biotechnol..

[23] Abyzov A., Tomasini L., Zhou B., Vasmatzis N., Coppola G., Amenduni M., Pattni R., Wilson M., Gerstein M., Weissman S. (2017). One Thousand Somatic SNVs per Skin Fibroblast Cell Set Baseline of Mosaic Mutational Load with Patterns That Suggest Proliferative Origin. Genome Res..

[24] Zhao T., Zhang Z.-N., Rong Z., Xu Y. (2011). Immunogenicity of Induced Pluripotent Stem Cells. Nature.

[25] Galipeau J., Sensébé L. (2018). Mesenchymal Stromal Cells: Clinical Challenges and Therapeutic Opportunities. Cell Stem Cell.

[26] Plaza Reyes A., Petrus-Reurer S., Padrell Sánchez S., Kumar P., Douagi I., Bartuma H., Aronsson M., Westman S., Lardner E., André H. (2020). Identification of Cell Surface Markers and Establishment of Monolayer Differentiation to Retinal Pigment Epithelial Cells. Nat. Commun..

[27] Ludlow K. (2020). Genetic Identity Concerns in the Regulation of Novel Reproductive Technologies. J. Law Biosci..

[28] Lovell-Badge R., Anthony E., Barker R.A., Bubela T., Brivanlou A.H., Carpenter M., Charo R.A., Clark A., Clayton E., Cong Y. (2021). ISSCR Guidelines for Stem Cell Research and Clinical Translation: The 2021 Update. Stem Cell Rep..

[29] Komor A.C., Kim Y.B., Packer M.S., Zuris J.A., Liu D.R. (2016). Programmable Editing of a Target Base in Genomic DNA without Double-Stranded DNA Cleavage. Nature.

[30] Anzalone A.V., Randolph P.B., Davis J.R., Sousa A.A., Koblan L.W., Levy J.M., Chen P.J., Wilson C., Newby G.A., Raguram A. (2019). Search-and-Replace Genome Editing without Double-Strand Breaks or Donor DNA. Nature.

[31] Huh D., Matthews B.D., Mammoto A., Montoya-Zavala M., Hsin H.Y., Ingber D.E. (2010). Reconstituting Organ-Level Lung Functions on a Chip. Science.

[32] Barberis M., Xie J. (2025). Towards a Unified Framework for Single-cell -omics-based Disease Prediction through AI. Clin. Transl. Med..

[33] Morrison M. (2017). “A Good Collaboration Is Based on Unique Contributions from Each Side”: Assessing the Dynamics of Collaboration in Stem Cell Science. Life Sci. Soc. Policy.

[34] Loring J.F., McDevitt T.C., Palecek S.P., Schaffer D.V., Zandstra P.W., Nerem R.M. (2014). A Global Assessment of Stem Cell Engineering. Tissue Eng. Part. A.

